# Synthesis and Pharmacological Evaluation of New 3,4-Dihydroisoquinolin Derivatives Containing Heterocycle as Potential Anticonvulsant Agents

**DOI:** 10.3390/molecules21121635

**Published:** 2016-11-29

**Authors:** Hong-Jian Zhang, Qing-Kun Shen, Chun-Mei Jin, Zhe-Shan Quan

**Affiliations:** Key Laboratory of Natural Resources and Functional Molecules of the Changbai Mountain, Affiliated Ministry of Education, College of Pharmacy, Yanbian University, Yanji 133002, China; zhjzhishixiang@163.com (H.-J.Z.); 2016001065@ybu.edu.cn (Q.-K.S.)

**Keywords:** synthesis, isoquinoline, anticonvulsant, MES, molecular docking

## Abstract

Two novel series of 3,4-dihydroisoquinolin with heterocycle derivatives (**4a**–**t** and **9a**–**e**) were synthesized and evaluated for their anticonvulsant activity using maximal electroshock (MES) test and pentylenetetrazole (PTZ)-induced seizure test. All compounds were characterized by IR, ^1^H-NMR, ^13^C-NMR, and mass spectral data. Among them, 9-(exyloxy)-5,6-dihydro-[1,2,4]triazolo[3,4-*a*]isoquinolin-3(2*H*)-one (**9a**) showed significant anticonvulsant activity in MES tests with an ED_50_ value of 63.31 mg/kg and it showed wide margins of safety with protective index (PI > 7.9). It showed much higher anticonvulsant activity than that of valproate. It also demonstrated potent activity against PTZ-induced seizures. A docking study of compound **9a** in the benzodiazepine (BZD)-binding site of γ-aminobutyric acid_A_ (GABA_A_) receptor confirmed possible binding of compound **9a** with the BZD receptors.

## 1. Introduction

Epilepsy is the second most common chronic neurological disorder, and can greatly affect the quality of life of patients. Nearly four out of five people with epilepsy live in low- and middle-income countries [1,2]. However, even in a high-income country, such as the US, epilepsy leads to 42,000 deaths every year [3]. The predominant type of epilepsy found in adult patients is temporal lobe epilepsy, which is generally caused by a primary precipitating injury that occurs during infancy; the incidence of epilepsy in elderly patients has been steadily increasing over the past 20 years [4,5]. Despite the development of several new anticonvulsants, one out of four epileptic seizures remain still inadequately treated [6]. Thus, we attempted to develop novel efficacious anticonvulsants to improve the quality of life for patients with epilepsy.

**Figure 1:**
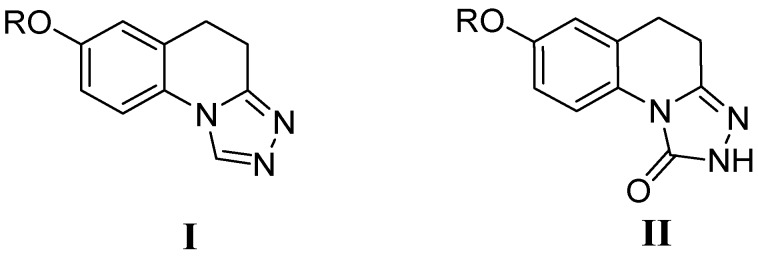
The structure of Lead compounds.

In our previous work, we reported that 7-alkoxyl-4,5-dihydro-[1,2,4]triazolo[4,3-*a*]quinoline derivatives (**I**) and 7-alkoxy-4,5-dihydro-[1,2,4]triazolo[4,3-*a*]quinoline-1(2*H*)-ones (**II**) exhibited potential anticonvulsant activity (Figure 1). In compound series **I**, compound 7-(4-fluorobenzyloxy)-4,5-dihydro-[1,2,4]triazolo[4,3-*a*]quinoline was found to be the most potent in protecting against MES-induced seizures, with an ED_50_ value of 11.8 mg/kg. In compound series **II**, 8-hexyloxy-4,5-dihydro-[1.2.4]triazole[4.3-*a*]quinoline-1-one was the most potent anticonvulsant, with an ED_50_ value of 17.17 mg/kg in the maximal electroshock (MES) test [7,8]. Moreover, numerous studies have previously shown that quinoline and isoquinoline derivatives have similar biological activity, such as antiproliferative [9], antihypertensive [10], analgesics [11], and antidepressant effects [12]. Therefore, we speculated that [1,2,4]triazolo[4,3-*a*]isoquinolines derivatives would have antiseizure activity. Two series of target compounds (**4a**–**t** and **9a**–**e**) were then designed and synthesized with 6-methoxy-2,3-dihydro-1*H*-inden-1-one as the starting material.

## 2. Results and Discussion

### 2.1. Chemistry

**Scheme 1:**
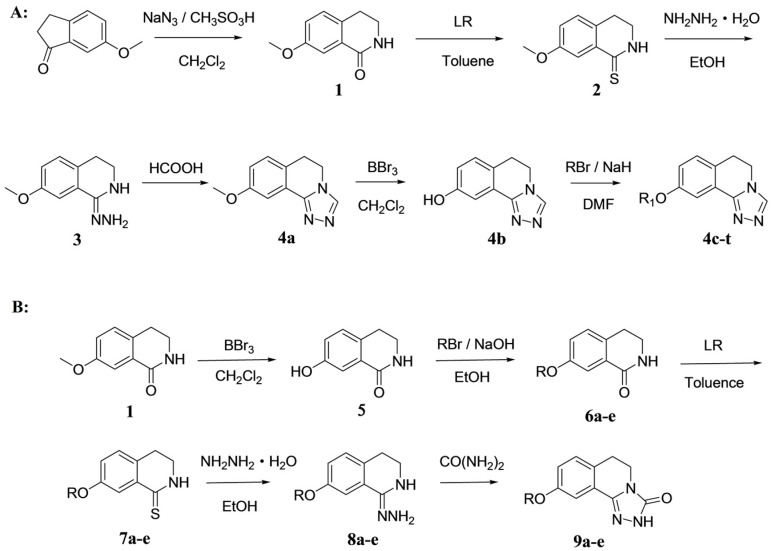
The synthesis of target compounds **4a**–**t** (**A**) and **9a**–**e** (**B**).

Target compounds **4a**–**t** and **5a**–**t** were synthesized using a previously reported method as depicted in Scheme 1A. 7-Methoxy-3,4-dihydroisoquinolin-1(2*H*)-one (compound **1**) was prepared by the Schmidt reaction, using 6-methoxy-2,3-dihydro-1*H*-inden-1-one with sodium azide in dichloromethane in the presence of methanesulfonic acid [13]. Compound **1** was reacted with Lawesson′s reagent in toluene to obtain 7-methoxy-3,4-dihydroisoquinoline-1(2*H*)-thione (compound **2**) [14]. Then, compound **2** was reacted with hydrazine hydrate in ethanol to produce 1-hydrazono-7-methoxy-1,2,3,4-tetrahydroisoquinoline (compound **3**) [15]. Compound **3** was further reacted with formic acid [16] to obtain 9-methoxy-5,6-dihydro-[1,2,4]triazolo[3,4-*a*]isoquinoline (compound **4a**). Compound **4b** was obtained from the above compounds by demethylation of boron tribromide. The compound was treated with alkyl halide or substituted benzyl halide in the presence of sodium hydride in *N*,*N*-dimethyl formamide to give compounds **4c**–**t**, respectively. The target compounds **9a**–**e** were synthesized using a slightly modified version of the methods used to synthesize the aforementioned compounds as shown in Scheme 1B. These five compounds were synthesized from compound **1** by an alkylation reaction, thionation, hydrazine group reaction, and a cyclization reaction [17]. We chose to modify the synthesis method in this way in order to obtain the bis-substituted product in the last step without altering the conditions.

### 2.2. Pharmacology

In our previous work, we reported that [1,2,4]triazolo[4,3-*a*]quinoline derivatives (**I**) and [1,2,4]triazolo[4,3-*a*]quinoline-1(2*H*)-ones (**II**) exhibited potential anticonvulsant activity [7,8]. Therefore, in this study, we evaluated the anticonvulsant activity of 9-alkoxy-5,6-dihydro-[1,2,4]triazolo[3,4-*a*]isoquinoline and 9-alkoxy-5,6-dihydro-[1,2,4] triazolo[3,4-*a*]isoquinolin-3(2*H*)-one, respectively.

The MES test and the rotarod test were carried out according to the methods described in the Antiepileptic Drug Development Program (ADD) of the National Institutes of Health (Bethesda, MD, USA) [18,19]. The anticonvulsant activities of all compounds were tested in KunMing mice (clean animal, weight: 18–22 g) purchased from the Laboratory of Animal Research, College of Pharmacy, Yanbian University. The methods were carried out in accordance with the Administration Rule of Laboratory Animals of China. All experimental protocols were approved by the Medical Ethics Committee of China.

As can be seen in Table 1, all compounds with the exception of **4a**, **4b**, **4g**, **4h**, **4o**, **4p**, **4r**, **4t**, **9c**, and **9e** showed anticonvulsant activity in the MES test at a dose of 100 mg/kg. In particular, compounds **4d**, **4e**, and **9a** had potent anticonvulsant activity and showed protective effects in three out of three mice. Additionally, eight compounds (**4c**, **4d**, **4i**, **4j**, **4k**, **4m**, **4n**, **4q**, and **9d**) exhibited protective effects in two out of three mice and four compounds (**4f**, **4l**, **4s**, and **9b**) exhibited protective effects in one out of three mice, respectively. However, only compounds **4e**, **9a**, and **9d** showed protective effects at 30 mg/kg in one out of three mice. Compared with the other compounds in this series, compound **4b** did not exhibit anticonvulsant activity. This may suggest that the substituent at 9-position plays a key role in anticonvulsant activity, which is consistent with the findings obtained with the previous model. In general, the activity of compounds containing a branched chain was better than that of benzyl-substituted compounds. With regard to the benzene ring of benzyl, the activity of compounds with electron-withdrawing groups was stronger than that of those with replaced by electronics groups. In compounds **4a** and **4c**–**h**, the length of the alkyl chain played an important role in the anticonvulsant activity of the derivatives. From compounds **4a** to **4e**, as the alkyl chain length increased, the anti-MES activity gradually increased, with compound **4e** being the most active. When the length of the alkyl chain increased from **4e** to **4h**, the anticonvulsant activity decreased. From **4i**–**4q**, four compounds (**4j**, **4k**, **4m**, and **4n**) showed no significant difference in activity when a halogen atom was substituted at positions 2 and 3 of the benzene ring. However, if a halogen atom was substituted at position 4, the activity of the compound reduced or abolished completely. Comparing compound **4r** with compound **4j**–**l**, we found that the electron-withdrawing capacity of –CF_3_ was capable of eliminating anticonvulsant activity.

Because of their better activity and lower neurotoxicity, compounds **4e**, **9a**, and **9d** were evaluated for ED_50_ and TD_50_ values in phase-II. As shown in Table 2, compound **4e** exhibited the highest anticonvulsant activity, with an ED_50_ value of 48.19 mg/kg; its activity was higher than that of valproate, but weaker than that of carbamazepine. Compared with compound **4e**, compound **9a** and **9d** showed a weaker anticonvulsant activity, with an ED_50_ value of 63.31 mg/kg and 68.16 mg/kg, respectively. Instead, they exerted wide margins of safety with PIs that were much higher than those of currently used drug valproate (PI_9a_ > 7.9, PI_9d_ = 5.6).

In series **I**, the compound 7-(heptyloxy)-4,5-dihydro-[1,2,4]triazolo[4,3-*a*]quinoline showed potent anticonvulsant activity with an ED_50_ of 13.5 mg/kg and PI of 2.2 [7]. Compared with compound **4e**, it had lower anticonvulsant activity and neurotoxicity. Compounds **9a** and **9d** showed lower anticonvulsant activity but higher neurotoxicity than compounds **3c** and **3f** did in series **II** [8] and compound **4f** and **4h**, as reported by Sun et al. [20]. As can be observed from compound **3c** (ED_50_ = 11.8 mg/kg) and **3f** (ED_50_ = 12.3 mg/kg) to compound **4f** (ED_50_ = 19.7 mg/kg) and **4h** (ED_50_ = 38 mg/kg), the same alkoxy when transformed from the 7-position to the 8-position weakens the anticonvulsant activity, suggesting that the substituted position of the alkoxy plays an important role in the compound′s potency. Compared with compounds **4f** and **4h**, **9a** and **9d** only differed in the position of the N atom, producing decreased anticonvulsant activity and higher neurotoxicity. Thus, it seems that for holistic biological activity, the position of the N atom may be a decisive factor.

In our previous study, we found that most compounds of series **I** and **II** exhibited activity against subcutaneous (sc) pentylenetetrazole (PTZ)-induced seizures. In the following study, we chose to investigate the effect of compound **9a** on scPTZ-induced seizures in mice. The results are shown in Table 3. Carbamazepine inhibited clonic seizures, tonic seizures, and death at rates of 0%, 100%, and 100%, respectively, while the corresponding rates for compound **9a** (0%, 90%, 40%) were lower. We concluded that although compound **9a** did not absolutely protect mice from tonic seizures induced by PTZ, it could reduce the incidence of tonic convulsion and mortality.

### 2.3. Docking Study

PTZ has been reported to produce seizures by inhibiting γ-aminobutyric acid (GABA) neurotransmission [21]. Benzodiazepines (BZDs) are highly potent anticonvulsants that are commonly used in clinical treatment [22]. The GABA_A_ receptor is regarded as one of the targets for development of antiepileptic drugs [23,24,25].

**Figure 2:**
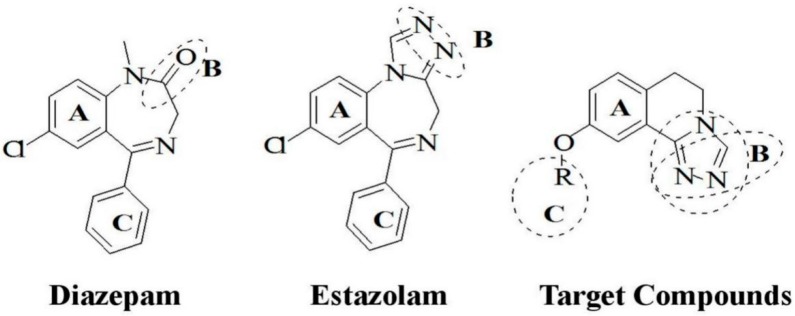
The structures of diazepam, estazolam, and the target compounds. (The dotted line was used to mark the special group).

Diazepam and estazolam express anticonvulsant activity through BZD receptors. As shown in Figure 2, three parts of their structures were considered to bind to the BZD receptors: an aromatic ring (A), a coplanar proton-accepting group (B), and second out-of-plane aromatic ring (C). The main parts of the structures of newly designed compounds were similar to theirs.

**Figure 3:**
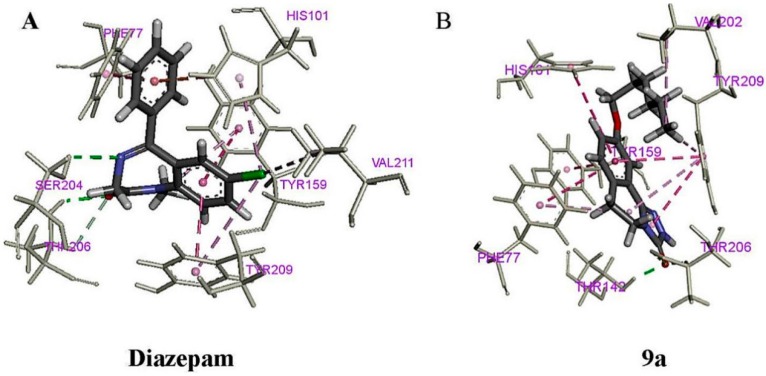
Images of docked complexes of GABA_A_ with diazepam and compound **9a** (thick color sticks) making interactions (color dotted lines) with residues at the BZD-binding site (silver-white sticks). (**A**) The complexes of GABA_A_ with diazepam; (**B**) The complexes of GABA_A_ with compound **9a**.

To confirm whether the anticonvulsant activity of compound **9a** is mediated through BZD receptors, a docking study was performed using Discovery Studio™ Server—Client (2017). As shown in Figure 3A, α1 Thr206, α1 Tyr 209, α1 His101, α1 Tyr159, and γ2 Phe77 are the most important residues in the binding mode of diazepam [23]. As shown in Figure 3B, the oxygen atom was responsible for conventional hydrogen bond (green dotted lines) interaction with α1 Thr206 and γ2 Thr142, and the length of bond was 2.95 and 1.88 Å. The benzene ring of compound **9a** was responsible for π-π (pink lines) interaction with α1 Tyr159, α1 Tyr 209, α1 His101 and γ2 Phe77, respectively. Besides, the oxygen atom in the alkoxy chain was interacted by carbon-hydrogen bond (laurel-green line) interaction with α1 Tyr159. Therefore, the results showed that the binding mode of compound **9a** was similar to that of diazepam in the BZD-binding pocket of the GABA_A_ receptor.

Indiplon and zopiclone are effective nonbenzodiazepine sedative hypnotic medications that act on benzodiazepine-binding sites on GABA_A_ receptors in the central nervous system [26,27]. Their derivatives also show higher binding affinities with GABA_A_ receptors; among these, two derivatives, 3-[2-(4-methylpiperazin-1-yl)-2-oxoethyl]-2-(5-nitropyridin-2-yl)iso-indolin-1-one (**III**) and *N*-(3-fluoro-propyl)-*N*-{3-[3-(thiophene-2-carbonyl)-pyrazolo[1,5-*a*]pyrimidine-7-yl]phenyl}acetamide (**IV**), showed the most pronounced efficacy at GABA_A_ receptors [28,29]. In the present study, we calculated the similarity between compounds **9a** and **9d**, and compounds **III** and **IV**. LibDock protocol was executed, and the top LibDockScore was preferred. Docking results are described in Table 4. The LibDockScore of compound **9a** was lower than compound **III** and **IV**, but higher than compound **9d**. Docking results suggest that compound **9a** has greater efficacy at the BZD-binding site of the GABA_A_ receptor.

Lipinski′s Rule of Five was accepted and known in the study of drug-likeness. Among them, polar surface area (PSA) is an important index to evaluate their absorption ability through membrane. Generally, the PSA value was hard to measure; Ertl et al. [30] claimed that the TPSA value could take place of the PSA value, coincidentally in 2000. To speculate their bioavailability, the five parameters were calculated using the Molinspiration online property calculation toolkit and the formula reported by Zhao et al. [31] in this test. As shown in Table 5, compound **9a** obeyed these parameters, so we inferred that their oral preparations would be suitable for epilepsy therapy.

## 3. Experimental Section

### 3.1. Chemistry

Melting points were determined in open capillary tubes and were uncorrected. IR spectra were recorded (in KBr) on IR Prestige-21 (PerkineElmer, Waltham, MA, USA). ^1^H-NMR and ^13^C-NMR spectra were measured on an AV-300 (Bruker, Switzerland), and all chemical shifts were given in ppm relative to TMS. High resolution mass spectra were measured on an MALDI-TOF/TOF mass spectrometer (Bruker Daltonik GmbH, Bremen, Germany). The chemicals were purchased from Aldrich Chemical Corporation.

#### 3.1.1. Synthesis of 7-Methoxy-3,4-dihydroisoquinolin-1(2*H*)-one (**1**)

6-Methoxy-1-indenone (5.0 g, 30 mmol) was dissolved in methylene chloride/methanesulfonic acid (*v*:*v* = 3:1, 15 mL), sodium azide (3.0 g, 45 mmol ) was added slowly into the mixture under the condition of ice bath. Then, the solution was allowed to warm to room temperature and stirred overnight. The reaction mixture was partitioned between methylene chloride and aqueous sodium hydroxide. The aqueous layer was extracted with methylene chloride. The combined organic layers were washed with water, dried over anhydrous MgSO_4_, and evaporated to dryness, and the resulting residue was purified using column chromatography with the mixture of ethyl acetate and petroleum ether (3:1) as the eluent.

Yield: 55%, m.p. 101–102 °C. ^1^H-NMR (CDCl_3_, 300 MHz) δ: 2.94 (t, 2H, *J* = 7.50 Hz, –CH_2_–), 3.52–3.58 (m, 2H, –CH_2_–), 3.85 (s, 3H, –OCH_3_), 6.38 (s, 1H, –CONH–), 7.01 (dd, 1H, *J*_1_ = 9.00 Hz, *J*_2_ = 3.00 Hz, Ar-H), 7.14 (d, 1H, *J* = 9.00 Hz, Ar-H), 7.60 (d, 1H, *J* = 3.00 Hz, Ar-H).

#### 3.1.2. Synthesis of 7-Methoxy-3,4-dihydroisoquinoline-1(2*H*)-thione (**2**)

To a solution of the compound **1** (2.0 g, 11.3 mmol) in toluene (40 mL) was added Lawesson’s reagent (2.7 g, 6.8 mmol). The mixture was refluxed for 4 h. After removing the solvent under reduced pressure, the residue was purified by silica gel column chromatography with dichloromethane to give a yellow solid.

Yield: 81%, m.p. 122–123 °C. ^1^H-NMR (CDCl_3_, 300 MHz) δ: 2.95 (t, 2H, *J* = 6.00 Hz, –CH_2_–), 3.49–3.56 (m, 2H, –CH_2_–), 3.88 (s, 3H, –OCH_3_), 7.02 (dd, 1H, *J*_1_ = 6.00 Hz, *J*_2_ = 3.00 Hz, Ar-H), 7.09 (d, 1H, *J* = 9.00 Hz, Ar-H), 8.08 (d, 1H, *J* = 3.00 Hz, Ar-H), 8.69 (s, 1H, –CONH–).

#### 3.1.3. Synthesis of 9-Methoxy-5,6-dihydro-[1,2,4]triazolo[3,4-*a*]isoquinoline (**4a**)

A solution of the compound **2** (1.5 g, 8.8 mmol), hydrazine hydrate (60%, 10.0 mmol), and ethanol (25 mL) was refluxed for 2 h with stirring under nitrogen. The reaction mixture was then poured into cold water, and a crude product (3) was filtrated. This product was allowed to transfer to the round-bottom flask, formic acid (20 mL) was added and stirred overnight at 100 °C. The mixture was poured into ice water. The obtained target compound (**4a**) was filtered, washed with water, dried, and purified by silica gel column chromatography using CH_2_Cl_2_/CH_3_OH (100:1) as the eluent.

Yield: 35%, m.p. 128–129 °C. ^1^H-NMR (CDCl_3_, 300 MHz) δ: 3.15 (t, 2H, *J* = 6.00 Hz, –CH_2_–), 3.89 (s, 3H, –OCH_3_), 4.25 (t, 2H, *J* = 6.00 Hz, –CH_2_–), 6.95–6.99 (dd, 1H, *J*_1_ = 9.00 Hz, *J*_2_ = 3.00 Hz, Ar-H), 7.23 (d, 1H, *J* = 9.00 Hz, Ar-H), 7.73 (s, 1H, Ar-H), 8.21 (s, 1H, –N=CH–). ^13^C-NMR (CDCl_3_, 75 MHz) δ: 27.38, 40.67, 66.66, 108.15, 118.01, 124.39, 129.13, 150.21, 155.83, 159.32, 163.47. IR (KBr, cm^−1^): 1525, 1231, 1034. ESI-HRMS calculated for C_11_H_12_N_3_O^+^ ([M + H]^+^): 202.0975; found: 202.0982.

#### 3.1.4. Synthesis of 5,6-Dihydro-[1,2,4]triazolo[3,4-*a*]isoquinolin-9-ol (**4b**)

To a solution of compound **4a** (1.0 g, 5.0 mmol) in anhydrous dichloromethane (20 mL), boron tribromide (25 mmol) was added dropwise and the mixture was stirred for 1 h at 0 °C and then allowed to warm to a room temperature for 2 h. Following the addition of 20 mL ice cold water, the off-white solid was appeared. The reaction continued to be stirred for 1 h, then compound **4b** was obtained by being filtered, washed with water and dried.

Yield: 35%, m.p. 276–278 °C. ^1^H-NMR (DMSO-*d*_6_, 75 MHz) δ: 3.15 (t, 2H, *J* = 6.00 Hz, –CH_2_–), 4.43 (t, 2H, *J* = 6.00 Hz, –CH_2_–), 7.03 (d, 1H, *J* = 9.00 Hz, Ar-H), 7.35 (d, 1H, *J* = 9.00 Hz, Ar-H), 7.38 (s, 1H, Ar-H), 9.59 (s, 1H, –N=CH–), 9.90 (s, 1H, –OH). ^13^C-NMR (DMSO-*d*_6_, 75 MHz) δ: 25.66, 42.72, 100.81, 120.03, 120.45, 125.70, 130.24, 142.91, 149.10, 156.80. IR (KBr, cm^−1^): 3255, 1589, 1223, 1042. ESI-HRMS calculated for C_10_H_10_N_3_O^+^ ([M + H]^+^): 188.0818; found: 188.0815.

#### 3.1.5. Synthesis of 9-Alkoxy-5,6-dihydro-[1,2,4]triazolo[3,4-*a*]isoquinoline (**4c**–**t**)

Compound **4b** (0.2 g, 1.1 mmol) was dissolved in anhydrous DMF (2 mL), alkyl bromide, or benzyl chloride derivatives (1.2 mmol) and NaH (0.15 g, 6.4 mmol) were added into this solution and stirred at a room temperature for 0.5–2 h. The mixture was poured into ice water, and then filtered, washed and dried. The compound (**4c**–**t**) was isolated and purified by silica gel column chromatography using CH_2_Cl_2_/CH_3_OH (100:1) as the eluent.

*9-(Pentyloxy)-5,6-dihydro-[1,2,4]triazolo[3,4-a]isoquinoline* (**4c**) Yield: 13.1%, m.p. 108–110 °C. ^1^H-NMR (CDCl_3_, 300 MHz) δ: 0.94 (t, 3H, *J* = 6.00 Hz, –CH_3_), 1.34–1.50 (m, 4H, –CH_2_–), 1.77–1.86 (m, 3H, *J* = 6.00 Hz, –CH_2_–), 3.13 (t, 2H, *J* = 6.00 Hz, –CH_2_–), 4.04 (t, 2H, *J* = 6.00 Hz, –CH_2_–), 4.24 (t, 2H, *J* = 6.00 Hz, –CH_2_–), 6.96 (dd, 1H, *J*_1_ = 6.00 Hz, *J*_2_ = 3.00 Hz, Ar-H), 7.21 (d, 1H, *J* = 9.00 Hz, Ar-H), 8.20 (s, 1H, –N=CH–). ^13^C-NMR (CDCl_3_, 75 MHz) δ: 14.04, 22.45, 27.54, 28.13, 28.87, 41.88, 68.42, 108.83, 118.28, 124.64, 124.75, 129.21, 142.08, 150.62, 158.83. IR (KBr, cm^−1^): 1522, 1219, 1013. ESI-HRMS calculated for C_15_H_20_N_3_O^+^ ([M + H]^+^): 258.1601; found: 258.1612.

*9-(Hexyloxy)-5,6-dihydro-[1,2,4]triazolo[3,4-a]isoquinoline* (**4d**) Yield: 18%, m.p. 78–80 °C. ^1^H-NMR (CDCl_3_, 300 MHz) δ: 0.91 (s, 3H, –CH_3_), 1.35 (d, 4H, *J* = 3.00 Hz, –CH_2_–), 1.47 (s, 2H, –CH_2_–), 1.75–1.87 (m, 2H, –CH_2_–), 3.13 (t, 2H, *J* = 6.00 Hz, –CH_2_–), 4.03 (t, 2H, *J* = 6.00 Hz, –OCH_2_–), 4.24 (t, 2H, *J* = 6.00 Hz, –CH_2_–), 6.96 (t, 1H, *J* = 6.00 Hz, Ar-H), 7.20 (d, 1H, *J* = 9.00 Hz, Ar-H), 7.69 (s, 1H, Ar-H), 8.21 (s, 1H, –N=CH–). ^13^C-NMR (CDCl_3_, 75 MHz) δ: 14.05, 22.60, 25.66, 27.54, 29.13, 31.55, 41.91, 68.43, 108.84, 118.27, 124.68, 124.77, 129.21, 142.36, 150.28, 158.83. IR (KBr, cm^−1^): 1518, 1217, 1016. ESI-HRMS calculated for C_16_H_22_N_3_O^+^ ([M + H]^+^): 272.1757; found: 272.1758.

*9-(Heptyloxy)-5,6-dihydro-[1,2,4]triazolo[3,4-a]isoquinoline* (**4e**) Yield: 30.3%, m.p. 89–90 °C. ^1^H-NMR (CDCl_3_, 300 MHz) δ: 0.90 (t, 3H, *J* = 6.00 Hz, –CH_3_), 1.31–1.51 (m, 8H, –CH_2_–), 1.75–1.85 (m, 2H, –CH_2_–), 3.12 (t, 2H, *J* = 6.00 Hz, –CH_2_–), 4.03 (t, 2H, *J* = 6.00 Hz, –OCH_2_–), 4.23 (t, 2H, *J* = 6.00 Hz, –CH_2_–), 6.94 (dd, 1H, *J*_1_ = 9.00 Hz, *J*_2_ = 3.00 Hz, Ar-H), 7.19 (d, 1H, *J* = 6.00 Hz, Ar-H), 7.69 (d, 1H, *J* = 3.00 Hz, Ar-H), 8.18 (s, 1H, –N=CH–). ^13^C-NMR (CDCl_3_, 75 MHz) δ: 13.91, 22.42, 25.76, 27.30, 28.84, 28.98, 31.59, 41.66, 68.21, 108.66, 117.90, 124.41, 124.67 129.04, 141.94, 150.37, 158.57. IR (KBr, cm^−1^): 1517, 1217, 1016. ESI-HRMS calculated for C_17_H_24_N_3_O^+^ ([M + H]^+^): 286.1914; found: 286.1919.

*9-(Octyloxy)-5,6-dihydro-[1,2,4]triazolo[3,4-a]isoquinoline* (**4f**) Yield: 28.9%, m.p. 76–78 °C. ^1^H-NMR (CDCl_3_, 300 MHz) δ: 0.89 (t, 3H, *J* = 6.00 Hz, –CH_3_), 1.29–1.48 (m, 10H, –CH_2_–), 1.75–1.85 (m, 2H, –CH_2_–), 3.12 (t, 2H, *J* = 6.00 Hz, –CH_2_–), 4.03 (t, 2H, *J* = 6.00 Hz, –OCH_2_–), 4.23 (t, 2H, *J* = 6.00 Hz, –CH_2_–), 6.95 (dd, 1H, *J*_1_ = 6.00 Hz, *J*_2_ = 3.00 Hz, Ar-H), 7.20 (d, 1H, *J* = 9.00 Hz, Ar-H), 7.69 (d, 1H, *J* = 3.00 Hz, Ar-H), 8.19 (s, 1H, –N=CH–). ^13^C-NMR (CDCl_3_, 75 MHz) δ: 13.10, 21.64, 24.96, 26.47, 28.14, 28.22, 28.31, 30.79, 40.84, 67.38, 107.78, 117.18, 123.55, 123.77, 128.20, 141.09, 149.57, 157.76. IR (KBr, cm^−1^): 1518, 1219, 1026. ESI-HRMS calculated for C_18_H_26_N_3_O^+^ ([M + H]^+^): 300.2070; found: 300.2075.

*9-(Nonyloxy)-5,6-dihydro-[1,2,4]triazolo[3,4-a]isoquinoline* (**4g**) Yield: 57.8%, m.p. 100–101 °C. ^1^H-NMR (CDCl_3_, 300 MHz) δ: 0.88 (t, 3H, *J* = 6.00 Hz, –CH_3_), 1.27–1.49 (m, 12H, –CH_2_–), 1.74–1.84 (m, 2H, –CH_2_–), 3.11 (t, 2H, *J* = 6.00 Hz, –CH_2_–), 4.02 (t, 2H, *J* = 6.00 Hz, –OCH_2_–), 4.23 (t, 2H, *J* = 6.00 Hz, –CH_2_–), 6.94 (dd, 1H, *J*_1_ = 6.00 Hz, *J*_2_ = 3.00 Hz, Ar-H), 7.19 (d, 1H, *J* = 9.00 Hz, Ar-H), 7.68 (d, 1H, *J* = 3.00 Hz, Ar-H), 8.18 (s, 1H, –N=CH–). ^13^C-NMR (CDCl_3_, 75 MHz) δ: 14.00, 17.83, 22.85, 25.86, 27.38, 29.05, 29.14, 29.25, 29.41, 31.75, 41.73, 68.20, 108.71, 118.04, 124.68, 129.09, 141.97, 150.45, 158.66. IR (KBr, cm^−1^): 1524, 1227, 1010. ESI-HRMS calculated for C_19_H_28_N_3_O^+^ ([M + H]^+^): 314.2227; found: 314.2230.

*9-(Decyloxy)-5,6-dihydro-[1,2,4]triazolo[3,4-a]isoquinoline* (**4h**) Yield: 69.9%, m.p. 89–91 °C. ^1^H-NMR (CDCl_3_, 300 MHz) δ: 0.88 (t, 3H, *J* = 6.00 Hz, –CH_3_), 1.27–1.50 (m, 14H, –CH_2_–), 1.74–1.84 (m, 2H, –CH_2_–), 3.11 (t, 2H, *J* = 6.00 Hz, –CH_2_–), 4.02 (t, 2H, *J* = 6.00 Hz, –OCH_2_–), 4.22 (t, 2H, *J* = 6.00 Hz, –CH_2_–), 6.94 (dd, 1H, *J*_1_ = 6.00 Hz, *J*_2_ = 3.00 Hz, Ar-H), 7.19 (d, 1H, *J* = 9.00 Hz, Ar-H), 7.68 (d, 1H, *J* = 3.00 Hz, Ar-H), 8.18 (s, 1H, –N=CH–). ^13^C-NMR (CDCl_3_, 75 MHz) δ: 14.02, 22.57, 25.88, 27.40, 29.06, 29.21, 29.26, 29.46, 29.47, 31.78, 41.75, 68.30, 108.74, 118.05, 124.49, 124. 69, 129.10, 142.00, 150.42, 158.67. IR (KBr, cm^−1^): 1518, 1219, 1003. ESI-HRMS calculated for C_20_H_30_N_3_O^+^ ([M + H]^+^): 328.2383; found: 328.2385.

*9-Benzyl-5,6-dihydro-[1,2,4]triazolo[3,4-a]isoquinoline* (**4i**) Yield: 14.9%, m.p. 150–152 °C. ^1^H-NMR (CDCl_3_, 300 MHz) δ: 3.12 (t, 2H, *J* = 6.00 Hz, –CH_2_–), 4.23 (t, 2H, *J* = 6.00 Hz, –CH_2_–), 5.14 (s, 2H, –OCH_2_–), 7.02 (dd, 1H, *J*_1_ = 9.00 Hz, J_2_ = 3.00 Hz, Ar-H), 7.21 (d, 1H, *J* = 9.00 Hz, Ar-H), 7.35–7.45 (m, 4H, Ar-H), 7.80 (dd, 1H, *J* = 6.00 Hz, Ar-H), 8.19 (s, 1H, –N=CH–). ^13^C-NMR (CDCl_3_, 75 MHz) δ: 27.46, 41.74, 70.15, 109.27, 118.33, 124.67, 125.24, 127.46, 128.00, 128.54, 129.25, 136.47, 142.02, 150.40, 158.30. IR (KBr, cm^−^^1^): 1520, 1205, 1020. ESI-HRMS calculated for C_17_H_16_N_3_O^+^ ([M + H]^+^): 278.1288; found: 278.1292.

*9-(2-Fluorobenzyloxy)-5,6-dihydro-[1,2,4]triazolo[3,4-a]isoquinoline* (**4j**) Yield: 57.2%, m.p. 137–139 °C. ^1^H-NMR (CDCl_3_, 300 MHz) δ: 3.12 (t, 2H, *J* = 6.00 Hz,–CH_2_–), 4.22 (t, 2H, *J* = 6.00 Hz, –CH_2_–), 5.18 (s, 2H, –OCH_2_–), 7.01 (dd, 1H, *J*_1_ = 6.00 Hz, *J*_2_ = 3.00 Hz, Ar-H), 7.05–7.22 (m, 3H, Ar-H), 7.26–7.35 (m, 1H, Ar-H), 7.49–7.53 (m, 1H, Ar-H), 7.81 (d, 1H, *J* = 3.00 Hz, Ar-H), 8.17 (s, 1H, –N=CH–). ^13^C-NMR (CDCl_3_, 75 MHz) δ: 27.33, 41.6, 63.97, 109.31, 115.10, 115.38, 117.86, 123.68, 124.11, 124.61, 125.51, 129.28, 129.60, 142.04, 150.24, 157.97, 161.99. IR (KBr, cm^−1^): 1515, 1213, 1016. ESI-HRMS calculated for C_17_H_15_FN_3_O^+^ ([M + H]^+^): 296.1194; found: 296.1198.

*9-(3-Fluorobenzyloxy)-5,6-dihydro-[1,2,4]triazolo[3,4-a]isoquinoline* (**4k**) Yield: 44.5%, m.p. 140–142 °C. ^1^H-NMR (CDCl_3_, 300 MHz) δ: 3.4 (t, 2H, *J* = 6.00 Hz, –CH_2_–), 4.24 (t, 2H, *J* = 6.00 Hz, –CH_2_–), 5.14 (s, 2H, –OCH_2_–), 7.03 (dd, 2H, *J*_1_ = 6.00 Hz, *J*_2_ = 3.00 Hz, Ar-H), 7.16–7.24 (m, 3H, Ar-H), 7.32–7.39 (m, 1H, Ar-H), 7.77 (d, 1H, *J* = 3.00 Hz, Ar-H), 8.20 (s, 1H, –N=CH–). ^13^C-NMR (CDCl_3_, 75 MHz) δ: 27.47, 41.69, 69.24, 109.17, 113.92, 114.21, 114.64, 114.92, 122.67, 124.69, 125.50, 129.33, 130.13, 139.12, 142.06, 150.30, 157.94, 164.49. IR (KBr, cm^−1^): 1522, 1211, 1014. ESI-HRMS calculated for C_17_H_15_FN_3_O^+^ ([M + H]^+^): 296.1194; found: 296.1196.

*9-(4-Fluorobenzyloxy)-5,6-dihydro-[1,2,4]triazolo[3,4-a]isoquinoline* (**4l**) Yield: 16.5%, m.p. 149–150 °C. ^1^H-NMR (CDCl_3_, 300 MHz) δ: 3.15 (t, 2H, *J* = 6.00 Hz, –CH_2_–), 4.26 (t, 2H, *J* = 6.00 Hz, –CH_2_–), 5.12 (s, 2H, –OCH_2_–), 7.03 (dd, 1H, *J*_1_ = 6.00 Hz, *J*_2_ = 3.00 Hz, Ar-H), 7.10 (t, 2H, *J* = 6.00 Hz, Ar-H), 7.24 (d, 2H, *J* = 9.00 Hz, Ar-H), 7.45 (q, 2H, Ar-H), 8.21 (s, 1H, –N=CH–). ^13^C-NMR (CDCl_3_, 75 MHz) δ: 27.53, 41.82, 69.55, 109.23, 115.41, 115.70, 118.46, 124.77, 125.45, 129.40, 132.28, 132.32, 142.15, 150.46, 158.19, 160.91, 164.18. IR (KBr, cm^−1^): 1514, 1209, 1012. ESI-HRMS calculated for C_17_H_15_FN_3_O^+^ ([M + H]^+^): 296.1194; found: 296.1201.

*9-(2-Chlorobenzyloxy)-5,6-dihydro-[1,2,4]triazolo[3,4-a]isoquinoline* (**4m**) Yield: 45.2%, m.p. 140–142 °C. ^1^H-NMR (CDCl_3_, 300 MHz) δ: 3*.14 (t, 2H, J* = 6.00Hz, –CH_2_–), 4.25 (t, 2H, *J* = 6.00 Hz, –CH_2_–), 5.23 (s, 2H, –OCH_2_–), 7.04 (dd, 1H, *J*_1_ = 9.00 Hz, *J*_2_ = 3.00 Hz, Ar-H), 7.22 (s, 1H, Ar-H), 7.27–7.33 (m, 2H, Ar-H), 7.40–7.43 (m, 1H, Ar-H), 7.57–7.40 (m, 1H, Ar-H), 7.83 (d, 1H, *J* = 3.00 Hz, Ar-H), 8.20 (s, 1H, –N=CH–). ^13^C-NMR (CDCl_3_, 75 MHz) δ: 27.37, 41.64, 67.26, 109.44, 117.88, 124.64, 125.49, 126.80, 128.66, 128.97, 129.26, 132.58, 134.14, 141.99, 150.23, 157.97. IR (KBr, cm^−1^): 1517, 1213, 1047. ESI-HRMS calculated for C_17_H_15_ClN_3_O^+^ ([M + H]^+^): 312.0898; found: 312.0903.

*9-(3-Chlorobenzyloxy)-5,6-dihydro-[1,2,4]triazolo[3,4-a]isoquinoline* (**4n**) Yield: 33.2%, m.p. 127–128 °C. ^1^H-NMR (CDCl_3_, 300 MHz) δ: 3.14 (t, 2H, *J* = 6.00 Hz, –CH_2_–), 4.24 (t, 2H, *J* = 6.00 Hz, –CH_2_–), 5.11 (s, 2H, –OCH_2_–), 7.02 (dd, 1H, *J*_1_ = 9.00 Hz, *J*_2_ = 3.00 Hz, Ar-H), 7.22 (dd, 1H, *J*_1_ = 9.00 Hz, *J*_2_ = 3.00 Hz, Ar-H), 7.31 (m, 3H, Ar-H), 7.45 (s, 1H, Ar-H), 7.77 (d, 1H, *J* = 3.00 Hz, Ar-H), 8.19 (s, 1H, –N=CH–). ^13^C-NMR (CDCl_3_, 75 MHz) δ: 27.38, 41.66, 69.17, 109.11, 118.16, 124.66, 125.26, 125.49, 127.23, 128.03, 129.32, 129.78, 134.38, 138.51, 142.04, 150.23, 157.89. IR (KBr, cm^−1^): 1512, 1211, 1012. ESI-HRMS calculated for C_17_H_15_ClN_3_O^+^ ([M + H]^+^): 312.0898; found: 312.0894.

*9-(4-Chlorobenzyloxy)-5,6-dihydro-[1,2,4]triazolo[3,4-a]isoquinoline* (**4o**) Yield: 51.3%, m.p. 166–168 °C. ^1^H-NMR (CDCl_3_, 300 MHz) δ: 3.13 (t, 2H, *J* = 6.00 Hz, –CH_2_–), 4.23 (t, 2H, *J* = 6.00 Hz, –CH_2_–), 5.10 (s, 2H, –OCH_2_–), 7.00 (dd, 1H, *J*_1_ = 9.00 Hz, *J*_2_ = 3.00 Hz, Ar-H), 7.22 (d, 1H, *J* = 9.00 Hz, Ar-H), 7.33–7.40 (m, 3H, Ar-H), 7.76 (d, 1H, *J* = 3.00 Hz, Ar-H), 8.19 (s, 1H, –N=CH–). ^13^C-NMR (CDCl_3_, 75 MHz) δ: 27.35, 41.64, 69.23, 109.15, 118.13, 124.62, 125.42, 128.62, 128.65, 133.65, 134.94, 142.02, 150.24, 157.90. IR (KBr, cm^−1^): 1522, 1212, 1016. ESI-HRMS calculated for C_17_H_15_ClN_3_O^+^ ([M + H]^+^): 312.0898; found: 312.0893.

*9-[(2,4-Dichlorobenzyl)oxy]-5,6-dihydro-[1,2,4]triazolo[3,4-a]isoquinoline* (**4p**) Yield: 51.6%, m.p. 128–130 °C. ^1^H-NMR (CDCl_3_, 300 MHz) δ: 3.15 (t, 2H, *J* = 6.00 Hz, –CH_2_–), 4.25 (t, 2H, *J* = 6.00 Hz, –CH_2_–), 5.18 (s, 2H, –OCH_2_–), 7.03 (dd, 1H, *J*_1_ = 9.00 Hz, *J*_2_ = 3.00 Hz, Ar-H), 7.23–7.30 (m, 2H, Ar-H), 7.44 (d, 1H, *J* = 3.00 Hz, Ar-H), 7.52 (d, 1H, *J* = 3.00 Hz, Ar-H), 7.81 (d, 1H, *J* = 3.00 Hz, Ar-H), 8.21 (s, 1H, –N=CH–). ^13^C-NMR (CDCl_3_, 75 MHz) δ: 27.40, 41.66, 66.69, 109.37, 117.88, 124.72, 125.69, 127.14, 129.10, 129.35, 129.46, 132.87, 133.15, 134.07, 142.04, 150.20, 157.73. IR (KBr, cm^−1^): 1520, 1223, 1012. ESI-HRMS calculated for C_17_H_15_Cl_2_N_3_O^+^ ([M + H]^+^): 346.0508; found: 346.0515.

*9-[(2,6-Dichlorobenzyl)oxy]-5,6-dihydro-[1,2,4]triazolo[3,4-a]isoquinoline* (**4q**) Yield: 59.8%, m.p. 168–170 °C. ^1^H-NMR (CDCl_3_, 300 MHz) δ: 3.15 (t, 2H, *J* = 6.00 Hz, –CH_2_–), 4.25 (t, 2H, *J* = 6.00 Hz, –CH_2_–), 5.35 (s, 2H, –OCH_2_–), 7.04 (dd, 1H, *J*_1_ = 9.00 Hz, *J*_2_ = 3.00 Hz, Ar-H), 7.22–7.39 (m, 4H, Ar-H), 7.88 (d, 1H, *J* = 3.00 Hz, Ar-H), 8.20 (s, 1H, –N=CH–). ^13^C-NMR (CDCl_3_, 75 MHz) δ: 27.47, 42.72, 66.46, 109.28, 118.50, 124.64, 125.64, 129.39, 129.31, 130.47, 131.73, 136.92, 142.05, 150.38, 159.40. IR (KBr, cm^−1^): 1520, 1219, 1010. ESI-HRMS calculated for C_17_H_15_Cl_2_N_3_O^+^ ([M + H]^+^): 346.0508; found: 346.0506.

*9-(3-(Trifluoromethyl)benzyloxy)-5,6-dihydro-[1,2,4]triazolo[3,4-a]isoquinoline* (**4r**) Yield: 47.7%, m.p. 116–118 °C. ^1^H-NMR (CDCl_3_, 300 MHz) δ: 3.15 (t, 2H, *J* = 6.00 Hz, –CH_2_–), 4.25 (t, 2H, *J* = 6.00 Hz, –CH_2_–), 5.19 (s, 2H, –OCH_2_–),7.04 (dd, 1H, *J*_1_ = 6.00 Hz, *J*_2_ = 3.00 Hz, Ar-H), 7.26 (q, 1H, Ar-H), 7.50–7.65 (m, 3H, Ar-H), 7.73 (s, 1H, Ar-H), 7.80 (d, 1H, *J* = 3.00 Hz, Ar-H), 8.21 (s, 1H, –N=CH–). ^13^C-NMR (CDCl_3_, 75 MHz) δ: 27.41, 41.68, 69.26, 109.14, 118.17, 123.89, 123.92, 124.72, 124.75, 125.63, 129.00, 129.38, 130.52, 137.52, 142.06, 150.28, 157.90. IR (KBr, cm^−1^): 1518, 1210, 1016. ESI-HRMS calculated for C_18_H_15_F_3_N_3_O^+^ ([M + H]^+^): 346.1162; found: 346.1165.

*9-(4-Methlybenzyloxy)-5,6-dihydro-[1,2,4]triazolo[3,4-a]isoquinoline* (**4s**) Yield: 61.3%, m.p. 163–164 °C. ^1^H-NMR (CDCl_3_, 300 MHz) δ: 2.36 (s, 3H, –CH_3_), 3.12 (t, 2H, *J* = 6.00 Hz, –CH_2_–), 4.23 (t, 2H, *J* = 6.00 Hz, –CH_2_–), 5.09 (s, 2H, –OCH_2_–), 7.01 (dd, 1H, *J*_1_ = 9.00 Hz, *J*_2_ = 3.00 Hz, Ar-H), 7.20 (d, 3H, *J* = 9.00 Hz, Ar-H), 7.34 (d, 2H, *J* = 6.00 Hz, Ar-H), 7.79 (d, 1H, *J* = 3.00 Hz, Ar-H), 8.18 (s, 1H, –N=CH–). ^13^C-NMR (CDCl_3_, 75 MHz) δ: 21.14, 27.44, 41.73, 70.10, 109.27, 118.30, 124.62, 125.17, 127.59, 129.22, 133.43, 137.79, 142.03, 150.40, 158.34. IR (KBr, cm^−1^): 1517, 1211, 1022. ESI-HRMS calculated for C_18_H_18_N_3_O^+^ ([M + H]^+^): 292.1444; found: 292.1445.

*2-[(5,6-Dihydro-[1,2,4]triazolo[3,4-a]isoquinolin-9-yloxy)methyl]benzonitrile* (**4t**) Yield: 35.2%, m.p. 166–167 °C. ^1^H-NMR (CDCl_3_, 300 MHz) δ: 3.14 (t, 2H, *J* = 6.00 Hz,–CH_2_–), 4.25 (t, 2H, *J* = 6.00 Hz, –CH_2_–), 5.30 (s, 2H, –OCH_2_–), 7.05 (dd, 1H, *J*_1_ = 9.00 Hz, *J*_2_ = 3.00 Hz, Ar-H),7.25 (d, 1H, *J* = 9.00 Hz, Ar-H), 7.43–7.48 (m, 1H, Ar-H), 7.61–7.73 (m, 3H, Ar-H), 7.81 (d, 1H, *J* = 3.00 Hz, Ar-H), 8.20 (s, 1H, –N=CH–). ^13^C-NMR (CDCl_3_, 75 MHz) δ: 27.33, 41.60, 67.82, 109.67, 111.26, 116.89, 117.60, 124.70, 125.96, 128.48, 128.57, 129.39, 132.87, 132.94, 139.83, 142.04, 150.09, 157.63. IR (KBr, cm^−1^): 1520, 1211, 1022. ESI-HRMS calculated for C_18_H_15_N_4_O^+^ ([M + H]^+^): 303.1240; found: 303.1243.

#### 3.1.6. Synthesis of 7-Alkoxy-3,4-dihydroisoquinolin-1(2*H*)-one (**6a**–**e**)

A solution of compound **6** (2.0 g, 12.3 mmol), alkyl bromide, or benzyl chloride derivatives (14.7 mmol), NaOH (0.5 g, 12.3 mol), and absolute ethanol (30 mL) were added to a round-bottomed flask, refluxed for 4–6 h. After the 2/3 solvent was removed, the residual solution was poured into ice-cold water. The residue was isolated and recrystallized in ethanol.

*7-(Hexyloxy)-3,4-dihydroisoquinolin-1(2H)-one* (**6a**) Yield: 75%, m.p. 51–52 °C. ^1^H-NMR (CDCl_3_, 300 MHz) δ: 0.92 (t, 3H, *J* = 7.50 Hz, –CH_3_), 1.32–1.35 (m, 4H, –CH_2_–), 1.42–1.51 (m, 2H, –CH_2_–), 1.75–1.84 (m, 2H, –CH_2_–), 2.94 (t, 2H, *J* = 6.00 Hz, –CH_2_–), 3.53–3.59 (m, 2H, –OCH_2_–), 4.05 (t, 2H, *J* = 7.50 Hz, –CH_2_–), 6.70 (s, 1H, –CONH–), 7.01 (dd, 1H, *J*_1_ = 9.00 Hz, *J*_2_ = 3.00 Hz, Ar-H), 7.13 (d, 1H, *J* = 6.00 Hz, Ar-H), 7.59 (d, 1H, *J* = 3.00 Hz, Ar-H).

*7-(Heptyloxy)-3,4-dihydroisoquinolin-1(2H)-one* (**6b**) Yield: 77%, m.p. 52–53 °C. ^1^H-NMR (CDCl_3_, 300 MHz) δ: 0.90 (t, 3H, *J* = 7.50 Hz, –CH_3_), 1.32–1.46 (m, 8H, –CH_2_–), 1.42–1.51 (m, 2H, –CH_2_–), 1.75–1.82 (m, 2H, –CH_2_–), 2.94 (t, 2H, *J* = 6.00 Hz, –CH_2_–), 3.53–3.58 (m, 2H, –CH_2_–), 3.54–3.58 (m, 2H, –OCH_2_–), 6.52 (s, 1H, –CONH–), 7.01 (dd, 1H, *J*_1_ = 9.00 Hz, *J*_2_ = 3.00 Hz, Ar-H), 7.13 (d,1H, *J* = 9.00 Hz, Ar-H), 7.59 (d, 1H, *J* = 3.00 Hz, Ar-H).

*7-(Octyloxy)-3,4-dihydroisoquinolin-1(2H)-one* (**6c**) Yield: 81%, m.p. 59–60 °C. ^1^H-NMR (CDCl_3_, 300 MHz) δ: 0.89 (t, 3H, *J* = 6.00 Hz, –CH_3_), 1.23–1.46 (m, 10H, –CH_2_–), 1.74–1.83 (m, 2H, –CH_2_–), 2.94 (t, 2H, *J* = 6.00 Hz, –CH_2_–), 3.53–3.58 (m, 2H, –CH_2_–), 4.01 (t, 2H, *J* = 6.00 Hz, –OCH_2_–), 6.60 (s, 1H, –CONH–), 7.01 (dd, 1H, *J*_1_ = 9.00 Hz, *J*_2_ = 3.00 Hz, Ar-H), 7.13 (d, 1H, *J* = 9.00 Hz, Ar-H), 7.59 (d, 1H, *J* = 3.00 Hz, Ar-H).

*7-(Benzyloxy)-3,4-dihydroisoquinolin-1(2H)-one* (**6d**) Yield: 78%, m.p. 122–123 °C. ^1^H-NMR (CDCl_3_, 300 MHz) δ: 2.96 (t, 2H, *J* = 6.00 Hz, –CH_2_–), 3.57 (m, 2H, –CH_2_–), 5.13 (s, 2H, –OCH_2_–), 6.38 (s, 1H, Ar-H), 7.08–7.17 (m, 2H, Ar-H), 7.34–7.48 (m, 5H, Ar-H), 7.72(s, 1H, –CONH–).

*7-(4-Chlorobenzyloxy)-3,4-dihydroisoquinolin-1(2H)-one* (**6e**) Yield: 76%, m.p. 129–131 °C. ^1^H-NMR (CDCl_3_, 300 MHz) δ: 2.96 (t, 2H, *J* = 6.00 Hz, –CH_2_–), 3.54–3.59 (m, 2H, –CH_2_–), 5.09 (s, 2H, –OCH_2_–), 7.08 (dd, 1H, *J*_1_ = 6.00 Hz, *J*_2_ = 3.00 Hz, Ar-H), 7.35–7.38 (m, 4H, Ar-H), 7.68 (d, 1H, *J* = 3.00 Hz, –CONH–).

#### 3.1.7. Synthesis of 7-Alkoxy-3,4-dihydroisoquinolin-1(2*H*)-thione (**7a**–**e**)

A solution of the compound **7a**–**e** (6.0 mmol) in toluene (30 mL) was added to Lawesson’s reagent (1.4 g, 6.8 mmol). The mixture was refluxed for 4 h. After removing the solvent under reduced pressure, the residue was purified by silica gel column chromatography with dichloromethane to give a yellow solid.

*7-(Hexyloxy)-3,4-dihydroisoquinoline-1(2H)-thione* (**7a**) Yield: 79%, m.p. 49–50 °C. ^1^H-NMR (CDCl_3_, 300 MHz) δ: 0.92 (t, 3H, *J* = 7.50 Hz, –CH_3_), 1.33–1.39 (m, 4H, –CH_2_–), 1.43–1.53 (m, 2H, –CH_2_–), 1.75–1.83 (q, 2H, –CH_2_–), 2.85 (t, 2H, *J* = 6.00 Hz, –CH_2_–), 3.51–3.57 (m, 2H, –CH_2_–), 3.73 (q, 2H, –CH_2_–), 4.05(t, 2H, –OCH_2_–), 7.00–7.10 (m, 2H, Ar-H), 8.07 (d, 1H, *J* = 3.00 Hz, Ar-H), 8.69 (s, 1H, –CSNH–).

*7-(Heptyloxy)-3,4-dihydroisoquinoline-1(2H)-thione* (**7b**) Yield: 68%, m.p. 59–60 °C. ^1^H-NMR (CDCl_3_, 300 MHz) δ: 0.91 (t, 3H, *J* = 6.00 Hz, –CH_3_), 1.33–1.50 (m, 8H, –CH_2_–), 1.76–1.85 (m, 2H, –CH_2_–), 2.95 (t, 2H, *J* = 3.00 Hz, –CH_2_–), 3.54 (s, 2H, –CH_2_–), 4.07 (t, 2H, *J* = 3.00 Hz, –CH_2_–), 7.00–7.10 (m, 2H, Ar-H), 8.07 (d, 1H, *J* = 3.00 Hz, Ar-H), 8.83 (s, 1H, –CSNH–).

*7-(Octyloxy)-3,4-dihydroisoquinoline-1(2H)-thione* (**7c**) Yield: 67%, m.p. 49–51 °C. ^1^H-NMR (CDCl_3_, 300 MHz) δ: 0.91 (t, 3H, *J* = 7.50 Hz, –CH_3_), 1.26–1.33 (m, 8H, –CH_2_–), 1.45–1.52 (m, 2H, –CH_2_–), 1.76–1.85 (m, 2H, –CH_2_–), 2.96 (t, 2H, *J* = 3.00 Hz, –CH_2_–), 3.51–3.57 (m, 2H, –CH_2_–), 4.05 (t, 2H, *J* = 3.00 Hz, –OCH_2_–), 7.02 (dd, 1H, *J*_1_ = 9.00 Hz, *J*_2_ = 3.00 Hz, Ar-H), 8.07 (d, 1H, *J* = 3.00 Hz, Ar-H), 8.39 (s, 1H, –CSNH–).

*7-(Benzyloxy)-3,4-dihydroisoquinoline-1(2H)-thione* (**7d**) Yield: 75%, m.p. 88–90 °C. ^1^H-NMR (CDCl_3_, 300 MHz) δ: 2.96 (t, 2H, *J* = 6.00 Hz, –CH_2_–), 3.51–3.55 (m, 4H, –CH_2_–), 5.15 (s, 2H, –OCH_2_–), 7.10 (t, 1H, *J* = 3.00 Hz, Ar-H), 7.32–7.49 (m, 5H, Ar-H), 8.20 (s, 1H, –CSNH–).

*7-(4-Chlorobenzyloxy)-3,4-dihydroisoquinoline-1(2H)-thione* (**7e**) Yield: 82%, m.p. 146–148 °C. ^1^H-NMR (CDCl_3_, 300 MHz) δ: 2.97 (t, 2H, *J* = 6.00 Hz, –CH_2_–), 3.51–3.58 (m, 4H, –CH_2_–), 5.12 (s, 2H, –OCH_2_–), 7.09 (t, 1H, *J* = 3.00 Hz, Ar-H), 7.36–7.43 (m, 4H, Ar-H), 8.17 (d, 1H, *J* = 3.00 Hz, Ar-H), 8.52 (s, 1H, –CSNH–).

#### 3.1.8. Synthesis of 9-Alkoxy-5,6-dihydro-[1,2,4]triazolo[3,4-*a*]isoquinolin-3(2*H*)-one (**9a**–**e**)

A crude product **9a**–**e** obtained from the reaction of compound **8a**–**e** (4.0 mmol) was allowed to transfer to a round-bottomed flask, and carbamide (3.6 g, 6.0 mmol) was added and heated up to 200 °C to melt it. The mixture was cooled and then washed with enough hot water to wash off the excess urea. The obtained target compound (**10a**–**e**) was filtered, dried, and purified by silica gel column chromatography using CH_2_Cl_2_/CH_3_OH (60:1) as the eluent.

*9-(Hexyloxy)-5,6-dihydro-[1,2,4]triazolo[3,4-a]isoquinolin-3(2H)-one* (**9a**) Yield: 17.2%, m.p. 186–188 °C. ^1^H-NMR (CDCl_3_, 300 MHz) δ: 0.90 (t, 3H, *J* = 7.50 Hz, –CH_3_), 1.31–1.35 (m, 4H, –CH_2_–), 1.41–1.50 (m, 2H, –CH_2_–), 1.47–1.83 (m, 2H, –CH_2_–), 1.74–1.83 (m, 2H, –CH_2_–), 3.05 (t, 2H, *J* = 6.00 Hz, –CH_2_–), 3.89 (t, 2H, *J* = 6.00 Hz, –OCH_2_–), 3.99 (t, 2H, *J* = 6.00 Hz, –CH_2_–), 6.95 (dd, 1H, *J*_1_ = 9.00 Hz, *J*_2_ = 3.00 Hz, Ar-H), 7.18 (d, 1H, *J* = 9.00 Hz, Ar-H), 7.38 (d, 1H, *J* = 3.00 Hz, Ar-H), 8.18 (s, 1H, –CONH–). ^13^C-NMR (CDCl_3_, 75 MHz) δ: 13.96, 22.52, 25.60, 25.74, 27.06, 29.06, 31.48, 37.50, 68.30, 68. 36, 107.89, 118.27, 118.33, 118.41, 124.15, 125.82, 129.38, 143.74, 154.96, 158.49. IR (KBr, cm^−1^): 3125 (NH), 1722 (C=O), 1506, 1218, 1051. ESI-HRMS calculated for C_16_H_20_N_3_O_2_^+^ ([M − H]^+^): 286.1556; found: 286.1919.

*9-(Heptyloxy)-5,6-dihydro-[1,2,4]triazolo[3,4-a]isoquinolin-3(2H)-one* (**9b**) Yield: 20%, m.p. 158–160 °C. ^1^H-NMR (DMSO-*d_6_*, 300 MHz) δ: 0.88 (t, 3H, *J* = 7.50 Hz, –CH_3_), 1.30–1.44 (m, 8H, –CH_2_–), 1.68–1.77 (m, 2H, –CH_2_–), 3.00 (t, 2H, *J* = 6.00 Hz, –CH_2_–), 3.72 (t, 2H, *J* = 6.00 Hz, –OCH_2_–), 4.01 (t, 2H, *J* = 6.00 Hz, –CH_2_–), 6.98 (dd, 1H, *J*_1_ = 9.00 Hz, *J*_2_ = 3.00 Hz, Ar-H), 7.26 (q, 2H, Ar-H), 11.69 (s,1H, –CONH–). ^13^C-NMR (DMSO-*d*_6_, 75 MHz) δ: 13.75, 21.92, 25.39, 26.44, 28.32, 28.63, 31.14, 36.86, 67.99,107.82, 117.38, 124.39, 126.48, 129.77, 142.63, 154.01, 157.89. IR (KBr, cm^−1^): 3161 (NH), 1675 (C=O), 1511, 1217, 1050. ESI-HRMS calculated for C_17_H_24_N_3_O_2_^+^ ([M + H]^+^): 302.1863; found: 302.1866.

*9-(Octyloxy)-5,6-dihydro-[1,2,4]triazolo[3,4-a]isoquinolin-3(2H)-one* (**9c**) Yield: 18%, m.p. 168–170 °C. ^1^H-NMR (DMSO-*d*_6_, 300 MHz) δ: 0.87 (t, 3H, *J* = 6.00 Hz, –CH_3_), 1.28–1.44 (m, 10H, –CH_2_–), 1.67–1.75 (m, 2H, –CH_2_–), 3.00 (t, 2H, *J* = 6.00 Hz, –CH_2_–), 3.72 (t, 2H, *J* = 6.00 Hz, –OCH_2_–), 4.00 (t, 2H, *J* = 6.00 Hz, –CH_2_–), 6.98 (dd, 1H, *J*_1_ = 9.00 Hz, *J*_2_ = 3.00 Hz, Ar-H), 7.26 (q, 2H, Ar-H), 11.59 (s, 1H, –CONH–). ^13^C-NMR (DMSO-*d*_6_, 75 MHz) δ: 13.74, 21.96, 25.43, 26.44, 28.63, 28.64, 31.15, 36.86, 67.99, 107.84, 117.36, 124.39, 126.46, 129.76, 142.62, 164.01, 167.89. IR (KBr, cm^−1^): 3151 (NH), 1709 (C=O), 1506, 1224, 1021. ESI-HRMS calculated for C_18_H_26_N_3_O_2_^+^ ([M + H]^+^): 316.2020; found: 316.2027.

*9-(Benzyloxy)-5,6-dihydro-[1,2,4]triazolo[3,4-a]isoquinolin-3(2H)-one* (**9d**) Yield: 21%, m.p. 180–182 °C. ^1^H-NMR (CDCl_3_, 300 MHz) δ: 3.05 (t, 2H, *J* = 7.50 Hz, –CH_2_–), 3.89 (t, 2H, *J* = 6.00 Hz, –CH_2_–), 5.11 (t, 2H, *J* = 6.00 Hz, –OCH_2_–), 7.03 (dd, 1H, *J*_1_ = 9.00 Hz, *J*_2_ = 3.00 Hz, Ar-H), 7.20 (d, 1H, *J* = 9.00 Hz, Ar-H), 7.33–7.49 (m, 5H, Ar-H), 9.75 (s, 1H, –CONH–). ^13^C-NMR (CDCl_3_, 75 MHz) δ: 27.08, 37.45, 70.26, 108.57, 118.60, 124.27, 126.36, 127.40, 128.01, 128.54, 129.50, 136.47, 143.77, 154.63, 158.17. IR (KBr, cm^−1^): 3143 (NH), 1715 (C=O), 1504, 1223, 1014. ESI-HRMS calculated for C_17_H_16_N_3_O_2_^+^ ([M + H]^+^): 294.1237; found: 294.1242.

*9-(4-Chlorobenzyloxy)-5,6-dihydro-[1,2,4]triazolo[3,4-a]isoquinolin-3(2H)-one* (**9e**) Yield: 25%, m.p. 250–252 °C. ^1^H-NMR (DMSO-*d*_6_, 300 MHz) δ: 3.00 (t, 2H, *J* = 6.00 Hz, –CH_2_–), 3.71 (t, 2H, *J* = 7.50, –CH_2_–), 5.17 (s, 2H, –OCH_2_–), 7.08 (dd, 1H, *J*_1_ = 9.00 Hz, *J*_2_ = 3.00 Hz, Ar-H), 7.32 (q, 2H, Ar-H), 7.44–7.51 (m, 4H, Ar-H), 11.81 (s, 1H, –CONH–). ^13^C-NMR (DMSO-*d*_6_, 75 MHz) δ: 26.32, 36.74, 68.49, 107.88, 117.68, 124.28, 126.96, 128.43, 129.33, 129.92, 132.36, 136.94, 142.46, 153.88, 157.13. IR (KBr, cm^−1^): 3164 (NH), 1726 (C=O), 1493, 1209, 1094. ESI-HRMS calculated for C_17_H_15_ClN_3_O_2_^+^ ([M + H]^+^): 328.0847; found: 328.0843.

### 3.2. Pharmacology

#### 3.2.1. Maximal Electroshock Seizure

At 0.5 h after the administration of the compounds, the activities were evaluated using the MES test [18,19]. Seizures were elicited in mice using a 60 Hz alternating current of 50 mA. The current was applied via corneal electrodes for 0.2 s. In phase-I screening—3 mice in one group—each compound was administered at the dose levels of 30 and 100 mg/kg as a preliminary investigation of anticonvulsant activity. In phase-II screening—5 mice in one-dose group—mice were given a range of intraperitoneal doses of the tested compound until at least three points were established in the range of 10%–90% seizure prevention. The ED_50_ was calculated by using the plot of these data.

#### 3.2.2. Neurotoxicity (NT) Screening

The neurotoxicities of the compounds were measured in mice by the rotarod test [18,19]. The number of animals used for one-dose group was consistent with the MES test. The mice were given i.p. injections of the test compounds. After 0.5 h animals were trained to stay on an accelerating rotarod of 1-inch diameter that rotates at 6 rpm for 1 min, respectively. If the tested mice did not fall down from the rod, we determine that neurotoxicity was negative; otherwise it was positive. Each test animal had three chances. The compound was identified as not showing neurotoxicity as long as there was one success.

#### 3.2.3. sc-PTZ-Induced Seizures

Mice were randomly divided into three groups, with 10 mice per group. The solution of PTZ (85 mg/kg) was performed in mice administration s.c. half an hour later after i.p. administration. Then, the animals were continued to be observed for 0.5 h, and the numbers of clonic seizure, tonic seizure, and deaths noted [18,32].

### 3.3. Molecular Modeling

In this study, we used the diazepam-bound GABA_A_ receptor model first reported by Ernst et al. [23,33]. The ligands and protein were prepared via Discovery Studio 2017 Server. LibDock protocol was executed and the top LibDockScore was preferred. The binding site was defined using current selection, diazepam. The input site sphere was built with a radius of 6.3083 in *x* = 43.6442, *y* = 43.8606 and *z* = 9.03233. And other parameters remained at the default status. The optimal docking result was analyzed using Discovery Studio 2017 Client (Discovery Studio V17.1.0.16143, NeoTrident Co., Ltd., Beijing, China).

## 4. Conclusions

In this study, we designed and synthesized two novel series of 3,4-dihydroisoquinolin derivatives (**4a**–**t** and **9a**–**e**) and evaluated their preliminary anticonvulsant activities by using the MES test. Neurotoxicity was examined using rotarod tests. Among the tested compounds, 9-(exyloxy)-5,6-dihydro-[1,2,4]triazolo[3,4-*a*]isoquinolin-3(2*H*)-one (**9a**) showed significant anticonvulsant activity in MES tests, with an ED_50_ value of 63.31 mg/kg, and it showed wide margins of safety for the protective index (PI > 7.9). It showed much higher anticonvulsant activity than that of valproate, and weaker than carbamazepine, unfortunately. Molecular docking simulations showed that the prototype compound **9a** can act as a BZD-receptor agonist. We therefore believe that its underlying mechanism may be related to GABA_A_ and that in vitro studies are necessary to confirm this. This compound could be developed into a potential oral preparation for epilepsy treatment. Although the compound **9a** showed potential anticonvulsant activity, further studies should be conducted to reveal the mechanism of its anticonvulsant-like effects. In general, with regard to anticonvulsant activity, isoquinoline derivatives containing heterocyclic moieties are weaker than quinoline derivatives containing heterocyclic moieties, while other activities require further study.

## Figures and Tables

**Table 1:**
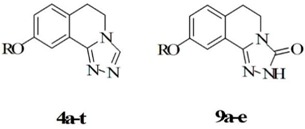
Phase-I evaluation of anticonvulsant activity in mice (i.p.).

Compound	R	MES ^a^		Toxicity	
		100	30	100	30
**4a**	–CH_3_	0/3	- ^b^	0/3	-
**4b**	–H	0/3	-	0/3	-
**4c**	*n*-C_5_H_11_	2/3	0/3	0/3	-
**4d**	*n*-C_6_H_13_	3/3	0/3	0/3	-
**4e**	*n*-C_7_H_15_	3/3	1/3	0/3	-
**4f**	*n-*C_8_H_17_	1/3	-	0/3	-
**4g**	*n*-C_9_H_19_	0/3	-	0/3	-
**4h**	*n*-C_10_H_21_	0/3	-	0/3	-
**4i**	–CH_2_C_6_H_5_	2/3	0/3	0/3	-
**4j**	–CH_2_C_6_H_4_ (*o*-F)	2/3	0/3	1/3	-
**4k**	–CH_2_C_6_H_4_ (*m*-F)	2/3	0/3	0/3	-
**4l**	–CH_2_C_6_H_4_ (*p*-F)	1/3	-	0/3	-
**4m**	–CH_2_C_6_H_4_ (*o*-Cl)	2/3	0/3	0/3	-
**4n**	–CH_2_C_6_H_4_ (*m*-Cl)	2/3	0/3	1/3	-
**4o**	–CH_2_C_6_H_4_ (*p*-Cl)	0/3	0/3	1/3	-
**4p**	–CH_2_C_6_H_3_ (*2,4*-2Cl)	0/3	0/3	0/3	-
**4q**	–CH_2_C_6_H_3_ (*2,6*-2Cl)	2/3	-	2/3	0/3
**4r**	–CH_2_C_6_H_4_ (*m*-CF_3_)	0/3	-	0/3	-
**4s**	–CH_2_C_6_H_4_ (*p*-CH_3_)	1/3	-	0/3	-
**4t**	–CH_2_C_6_H_4_ (*o*-CN)	0/3	-	0/3	-
**9a**	*n*-C_6_H_13_	3/3	1/3	0/3	-
**9b**	*n*-C_7_H_15_	1/3	-	0/3	-
**9c**	*n*-C_8_H_17_	0/3	-	0/3	-
**9d**	–CH_2_C_6_H_5_	2/3	1/3	0/3	-
**9e**	–CH_2_C_6_H_4_ (*p*-Cl)	0/3	-	0/3	-

**^a^** Maximal electroshock test (number of animals protected/number of animals tested), the number of mice is three; The dose measured in mg/kg; ^b^ Not tested.

**Table 2:** Phase-II quantitative anticonvulsant evaluation in mice (i.p.).

Compound	ED_50_^a^ (MES, mg/kg)	TD_50_^b^ (mg/kg)	PI ^c^
**4e**	48.19 (33.11–70.15) ^d^	163.26 (142.88–186.64)	3.4
**9a**	63.31 (46.17–86.82)	>500	>7.9
**9d**	68.16 (53.74–86.44)	378.62 (322.56–444.00)	5.6
Valproate	272 (247–338)	426 (369–450)	1.6
Carbamazepine	11.8 (8.5–16.4)	71.6 (45.9–153)	8.1

^a^ ED_50_: median effective dose affording anticonvulsant protection in 50% of animals, the dose is measured in mg/kg; ^b^ TD_50_: median toxic dose eliciting minimal neurological toxicity in 50% of animals, the dose is measured in mg/kg; ^c^ PI: protective index (TD_50_/ED_50_); ^d^ 95% confidence intervals given in parentheses.

**Table 3:** Effect of compound **9a** on scPTZ-induced seizures in mice (i.p.).

Compound	Doses (mg/kg)	Clonic Seizures (%) ^a^	Tonic Seizures (%)	Lethality (%)
DMSO	-	100	100	90
**9a**	100	100	10	60
Carbamazepine	50	100	0	0

^a^ The number of mice with clonic seizures, tonic seizures, and deaths/the number of animals tested ×100%.

**Table 4:**
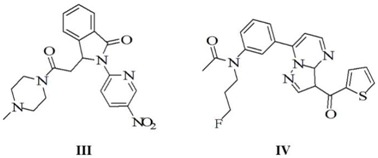
Docking results of compounds **9a**, **9d**, **III,** and **IV** by the protocol of LibDock.

Score Sorting	Compounds	LibDockScore
1	**III**	127.219
2	**IV**	120.181
3	**9a**	118.06
4	**9d**	118.036

**Table 5:** Pharmacokinetic parameters of compound **9a**.

Compound	MW ^a^ (< 500)	HBA ^b^ (< 10)	HBD ^c^ (≤ 5)	TPSA ^d^	%ABS ^e^
**9a**	287.36	5	1	59.92	88.33

^a^ MW: molecular weight; ^b^ HBA: number of hydrogen bond acceptors; ^c^ HBD: number of hydrogen bond donors; ^d^ TPSA: topological polar surface area; ^e^ %ABS: percentage of absorption.
